# Pretreatment Frequency of Circulating Th17 Cells and FeNO Levels Predicted the Real-World Response after 1 Year of Benralizumab Treatment in Patients with Severe Asthma

**DOI:** 10.3390/biom13030538

**Published:** 2023-03-15

**Authors:** Yuuki Sandhu, Norihiro Harada, Hitoshi Sasano, Sonoko Harada, Shoko Ueda, Tomohito Takeshige, Yuki Tanabe, Ayako Ishimori, Kei Matsuno, Sumiko Abe, Tetsutaro Nagaoka, Jun Ito, Asako Chiba, Hisaya Akiba, Ryo Atsuta, Kenji Izuhara, Sachiko Miyake, Kazuhisa Takahashi

**Affiliations:** 1 Department of Respiratory Medicine, Juntendo University Faculty of Medicine and Graduate School of Medicine, Tokyo 113-8421, Japan; 2 Research Institute for Diseases of Old Ages, Juntendo University Faculty of Medicine and Graduate School of Medicine, Tokyo 113-8421, Japan; 3 Atopy (Allergy) Research Center, Juntendo University Faculty of Medicine and Graduate School of Medicine, Tokyo 113-8421, Japan; 4 Department of Immunology, Juntendo University Graduate School of Medicine, Tokyo 113-8421, Japan; 5 Division of Medical Biochemistry, Department of Biomolecular Sciences, Saga Medical School, Saga 849-8501, Japan

**Keywords:** asthma, benralizumab, FeNO, Th17 cells, real-world setting

## Abstract

Benralizumab treatment reduces exacerbations and improves symptom control and quality of life in patients with severe eosinophilic asthma. However, the determination of biomarkers that predict therapeutic effectiveness is required for precision medicine. Herein, we elucidated the dynamics of various parameters before and after treatment as well as patient characteristics predictive of clinical effectiveness after 1 year of benralizumab treatment in severe asthma in a real-world setting. Thirty-six patients with severe asthma were treated with benralizumab for 1 year. Lymphocyte subsets in peripheral blood samples were analyzed using flow cytometry. Treatment effectiveness was determined based on the ACT score, forced expiratory volume in 1 s (FEV1), and the number of exacerbations. Benralizumab provided symptomatic improvement in severe asthma. Benralizumab significantly decreased peripheral blood eosinophil and basophil counts and the frequencies of regulatory T cells (Tregs), and increased the frequencies of Th2 cells. To our knowledge, this is the first study to show benralizumab treatment increasing circulating Th2 cells and decreasing circulating Tregs. Finally, the ROC curve to discriminate patients who achieved clinical effectiveness of benralizumab treatment revealed that the frequency of circulating Th17 cells and FeNO levels might be used as parameters for predicting the real-world response of benralizumab treatment in patients with severe asthma.

## 1. Introduction

Asthma affects over 300 million people worldwide, and there are more than 10 million people with asthma in Japan, of which approximately 10% of them have intractable asthma with symptoms that cannot be controlled by existing treatments. The medical costs for these patients account for the majority of the total medical costs associated with asthma. Asthma is one of the most common chronic diseases characterized by variable airflow limitation and bronchial hyperresponsiveness [1,2,3]. Of the various asthma phenotypes/endotypes, eosinophilic asthma affects more than 50% of patients with asthma [4,5]. Eosinophilic asthma responds well to treatment with inhaled corticosteroids (ICS) because steroids induce the apoptosis of eosinophils [6,7]. However, the effect of ICS on asthma is limited because some patients who develop the most severe clinical phenotype of eosinophilic asthma also have steroid-resistant refractory asthma. Biologics that are expected to have an additional effect on existing treatments for intractable asthma have been developed, with four of these agents being approved for use in Japan [8]. Among them, benralizumab, a humanized anti-IL-5 receptor α subunit monoclonal IgG1 antibody, depletes eosinophils by antibody-dependent cellular cytotoxicity. A major study evaluating benralizumab in patients with moderate to severe asthma found reduced exacerbations, improved lung function, and reduced use of oral corticosteroids (OCS) [9,10,11]. Benralizumab therapy reduces eosinophils in the peripheral blood and airway mucosa, especially by depleting eosinophils in the peripheral blood [11,12].

Based on previous studies, benralizumab has been used in patients with refractory asthma whose asthma symptoms cannot be controlled by existing therapies. Although high peripheral blood eosinophil levels are known to be a biomarker for predicting the effects of benralizumab treatment, a certain number of patients with asthma have been shown to respond to benralizumab, even with low levels of peripheral blood eosinophils [13,14]. Therefore, new biomarkers are needed to help predict the effectiveness of benralizumab therapy. Because biologics are expensive, extracting response cases before administration is important from both the perspective of health economics and future stratified medicine. In this study, we investigated whether benralizumab treatment was associated with peripheral blood immunocompetent cells and conducted an extensive study to identify important biomarkers for use in appropriately elucidating the effectiveness of benralizumab treatment.

## 2. Materials and Methods

### 2.1. Study Subjects

This study was a prospective observational study that enrolled severe asthma patients with newly prescribed benralizumab from March 2018 to May 2019. Patients who had severe asthma and were aged 20 years or older, whose asthma symptoms and asthma exacerbations requiring OCS could not be controlled by the existing treatment options despite treatment with high-dose ICS plus long-acting β2 agonists with another controller, and who required benralizumab treatment in the insurance medical treatment were recruited from our outpatient clinic at Juntendo University Hospital (Tokyo, Japan). Asthma was diagnosed based on a clinical history of episodic symptoms with airflow limitation and by either a variation in pulmonary function monitored by forced expiratory volume in 1 s (FEV1) or peak expiratory flow according to the Global Initiative for Asthma (GINA) guidelines [15]. Patients with any of the following criteria were excluded: (1) a diagnosis of eosinophilic granulomatosis with polyangiitis, interstitial pneumonia, infectious disease, or cancer; (2) those administering other antibody preparations; (3) cases that were judged as inappropriate by the study investigators; (4) cases under treatment with omalizumab and mepolizumab with <1 month of the last dose and cases under treatment with other biologics. The present study was reviewed and approved by the Juntendo University Research Ethics Committee (Tokyo, Japan). Written informed consent was obtained from each patient prior to participation in the study. The study was registered in the UMIN Clinical Trial Registry (UMIN000031905) on 23 March 2018 (http://www.umin.ac.jp/ (accessed on 25 March 2022)).

The asthma control test (ACT), pulmonary function tests, oscillometry (also known as the forced oscillation technique), measurement of fractional exhaled nitric oxide (FeNO) levels, and blood sampling were performed at the date of initial administration of benralizumab, 4 months, 8 months, and 1 year after administration. FeNO levels were measured in accordance with the American Thoracic Society recommendations at a constant flow of 0.05 L/s against an expiratory resistance of 20 cm water with an electrochemical hand-held NO analyzer (NIOX VERO^®^; Aerocrine AB, Solna, Sweden).

### 2.2. Definition for Responders

Patients were classified as responders and super-responders according to changes in ACT score, lung function, and asthma exacerbations with reference to previous studies [16,17,18,19,20,21,22]. A responder with benralizumab treatment was defined as meeting two of the following three criteria after 1 year of treatment with benralizumab without a significant deterioration in any other criterion: (1) Improvement in ACT score of at least 3 points (including patients who achieved an ACT score of 25 points) and an increase in ACT score of at least 3 points, which was previously suggested as the minimal clinically important difference [23,24]. (2) Reduction in the number of asthma exacerbations (including patients who had no exacerbations before and after treatment). (3) Improvement in FEV1 of at least 100 mL [22,25]. The following criteria were considered to be associated with significant deterioration: (a) a decreased ACT score of at least 3 points; (b) an increase in the number of exacerbations; (c) a decrease in FEV1 of at least 100 mL. A super-responder was also defined as meeting all three of the above criteria without showing any significant deterioration.

### 2.3. Quantification of Circulating Lymphocyte Frequency

Flow cytometry analysis was conducted as previously described [26,27]. Briefly, peripheral venous blood samples were collected in heparin-containing tubes, and PBMCs (3 × 10^6^/well) were purified by density-gradient centrifugation using Ficoll–Paque Plus solution (Cytiva, Tokyo, Japan). The cells were stained with different combinations of the appropriate antibodies for 30 min at 4 °C. The following surface marker antibodies were used in this study: anti-CD3-APC-H7, anti-CD4-FITC, anti-CD19-FITC, anti-CD56-PE-CF594, anti-CD117 (c-Kit)-PE-CF594 (BD Biosciences, San Jose, CA, USA), anti-T cell antigen receptor (TCR)-Pan-γδ-FITC, anti-TCR-Pan-γδ-PE (Beckman Coulter, Miami, FL, USA), anti-BDCA2-FITC, anti-CD1a-FITC, anti-CD3-FITC, anti-CD11c-FITC, anti-CD14-FITC, anti-CD25-PE, anti-CD34-FITC, anti-CD123-FITC, anti-CD127 (IL-7Rα)-Brilliant Violet 605, anti-CD161-PerCPCy5.5, anti-CD183 (CXCR3)-APC, anti-CD194 (CCR4)-Brilliant Violet 510, anti-CD196 (CCR6)-PerCPCy5.5, anti-CD294 (CRTH2)-Brilliant Violet 421, anti-FCɛR1-FITC, anti-Vα7.2- Brilliant Violet 605 (BioLegend, San Diego, CA, USA), and anti-hCD1d tetramer loaded with PBS-57-APC (NIH tetramer core facility at Emory University). Negative lineage markers (Lin^−^) were defined as CD1a^−^, CD3^−^, CD11c^−^, CD14^−^, CD19^−^, CD34^−^, TCRγδ^−^, CD123^−^, BDCA2^−^, and FCɛR1^−^. Th1 cells were identified as CD3^+^, CD4^+^, CCR4^−^, CCR6^−^, and CXCR3^+^ cells; Th2 cells as CD3^+^, CD4^+^, CCR4^+^, CCR6^−^, and CXCR3^−^ cells; Th17 cells as CD3^+^, CD4^+^, CCR4^+^, CCR6^+^, and CXCR3^−^ cells; Tregs as CD3^+^, CD4^+^, CD25^+^, and CD127^−^ cells; natural killer (NK) T (NKT) cells as CD3^+^ and CD1d/PBS-57 tetramer^+^ cells; γδT cells as CD3^+^ and TCRγδ^+^ cells; mucosal-associated invariant T (MAIT) cells as CD3^+^, Vα7.2 TCR^+^, and CD161^high^ cells; NK cells as CD3^−^ and CD56^+^ cells; ILC1 as Lin^−^, CD127^+^, CD161^+^, CD117^−^, and CRTH2^−^ cells; ILC2 as Lin^−^, CD127^+^, CD161^+^, and CRTH2^+^ cells; ILC3 as Lin^−^, CD127^+^, CD161^+^, CD117^+^, and CRTH2^−^ cells. Dead cells were identified by using the Zombie Fixable Viability Kit (BioLegend), followed by doublet exclusion on forward scatter and side scatter. After overnight fixation, the cells were analyzed by using a fluorescence-activated cell sorting (FACS) LSRFortessa cell analyzer (BD Biosciences). The FACS data were evaluated using FlowJo software (version 9; BD Biosciences).

### 2.4. Quantification of Serum Cytokine and Chemokine Levels

The sera of patients were collected after density-gradient centrifugation of blood samples, frozen at −80 °C, and assayed using the MILLIPLEX multiplex assay following the manufacturer’s instructions (Merck Millipore, Burlington, MA, USA). The assay-working range was determined between the lower limit of quantification and the upper limit of quantification (Supplementary Table S1). Serum periostin levels were measured using ELISA (Shino test, Kanagawa, Japan) as previously described [28]. Serum tenascin-C and regulated on activation normal T cell expressed and secreted (RANTES) were simultaneously quantified in thawed serum using the human tenascin-C ELISA kit (IBL, Gunma, Japan) and the human RANTES ELISA kit (R&D Systems, Minneapolis, MN, USA), respectively.

### 2.5. Statistical Analysis

Sample normality was examined using the D’Agostino–Pearson test. Differences in parameters between populations were analyzed for significance using Welch’s *t*-test, the paired *t* test, the Mann–Whitney *U* test, Wilcoxon’s signed-rank test, and Fisher’s exact test as appropriate. Comparisons between multiple groups were made by Friedman’s test with Dunn’s multiple comparisons test. ROC curve analyses were performed to differentiate between responders and non-responders to benralizumab. For correlation between variables, the Pearson’s correlation coefficient and Spearman’s rank correlation coefficient were used where appropriate. Differences were statistically significant when *p* values were <0.05. Statistical analyses were performed using GraphPad Prism version 8 software (GraphPad Software, San Diego, CA, USA).

## 3. Results

### 3.1. Baseline Characteristics

Thirty-six patients with severe asthma that was uncontrolled by existing treatment regimens, including who had an ACT score less than 20 points on conventional therapy or who had at least one exacerbation requiring oral corticosteroids per year, were enrolled and treated with benralizumab. Adverse events following the initial administration of benralizumab were observed in three patients, including a patient who had anaphylaxis, a patient who was suspected of having anaphylaxis, and a patient who had fever. In addition to the exclusion of the three aforementioned patients who developed adverse events, one patient withdrew consent and one patient had to discontinue the study due to exacerbation of allergic bronchial pulmonary aspergillosis and lower respiratory bacterial infection. Consequently, 31 patients were enrolled for the entire duration of the study. The baseline characteristics are shown in Table 1 and Table 2. The mean (±standard deviation) age of the patients was 54.3 ± 13.5 years (Table 1). Female patients, patients on regular OCS, patients who were treated with omalizumab, and those treated with mepolizumab prior to the study were 22 (71%), 3 (10%), 4 (13%), and 14 (45%), respectively (Table 1). The median daily dose of ICS was 1000 µg, inclusive of four patients who could not administer high-dose ICS due to hoarseness side-effects. Of the three patients with regular OCS, one received 1 mg of daily PSL and the other two received 5 mg of daily PSL. The median duration of asthma and duration of prior treatment with biologics (omalizumab or mepolizumab) was 16 years (interquartile range 8–26) and 604.5 days (426.5–690.0), respectively (Table 1). The effectiveness of previous biologics, except for two patients, was evaluated after at least 4 months of biologics treatment. Of the two patients that switched to benralizumab within 4 months of previous biologics treatment, one was discontinued omalizumab due to a side-effect, and the other was switched to benralizumab immediately after marketing because he had a request and did not recognize an effect of mepolizumab treatment.

Abbreviations for all tables: ACT, asthma control test; ABPA, allergic bronchopulmonary aspergillosis; AERD, aspirin-exacerbated respiratory disease; BMI, body mass index; FeNO, fractional exhaled nitric oxide; FP, fluticasone propionate; FVC, forced vital capacity; FEV_1_%, forced expiratory volume in 1 s/forced vital capacity; FEV_1_, forced expiratory volume in 1 s; ICS, inhaled corticosteroid; IFN-γ, interferon-gamma; IgE, immunoglobulin E; IL, interleukin; ILC, innate lymphoid cell; MAIT, mucosal associated invariant T; MCP, monocyte chemotactic protein; MIP, macrophage inflammatory protein; NK, natural killer; γδT, gamma delta T; NKT, natural killer T; RANTES, regulated on activation normal T cell expressed and secreted; SD, standard deviation; Th, helper T; Treg, regulatory T.

The mean FEV_1_%, which was calculated as FEV_1_/forced vital capacity (FVC), and the median (interquartile) peripheral blood eosinophil counts were 70.8 ± 17.6% and 80/μL (32–313), respectively (Table 2).

### 3.2. Changes in Each Parameter 1 Year after Benralizumab Treatment

**Figure 1:**
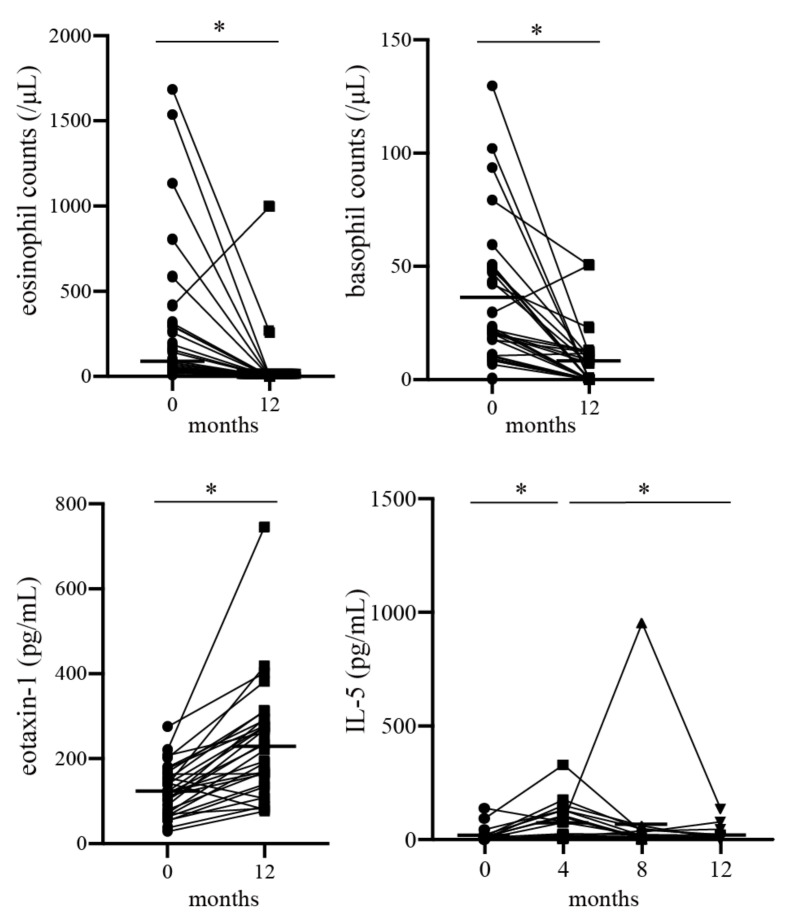
Changes in parameters from baseline to 12 months after treatment with benralizumab. Circulating eosinophil and basophil counts reduced significantly after benralizumab treatment. Serum eotaxin-1 levels and serum IL-5 levels increased significantly over the 1 year period and up to 4 months, respectively. Bars indicate median values. * *p* < 0.05.

After one year of benralizumab treatment, 18 (58%) of the 31 patients showed improved ACT scores of at least 3 points (known as the minimal clinically important difference [24]) or achieved total control. The comparison of initial data with data after 1 year of treatment with benralizumab was analyzed in 30 cases, excluding one case that was discontinued due to the worsening of asthma symptoms 6 months after the initiation of benralizumab treatment. The number of asthma exacerbations and unscheduled visits from worsening asthma decreased significantly, although the number of hospitalizations did not change. Benralizumab treatment significantly increased the ACT score, percent predicted FEV_1_ (%FEV_1_), FEV_1_% in pulmonary function parameters, and serum eotaxin-1 levels, but significantly decreased peripheral blood eosinophil and basophil counts (Table 3 and Figure 1). Although the serum levels of IL-5 did not change after 1 year of benralizumab treatment, they were significantly increased at 4 months and then significantly decreased at 12 months compared to 4 months (Table 3 and Figure 1). Because serum IL-4, IL-13, and macrophage inflammatory protein (MIP)-1α levels were below the detection limit, they were excluded from the analysis.

**Figure 2:**
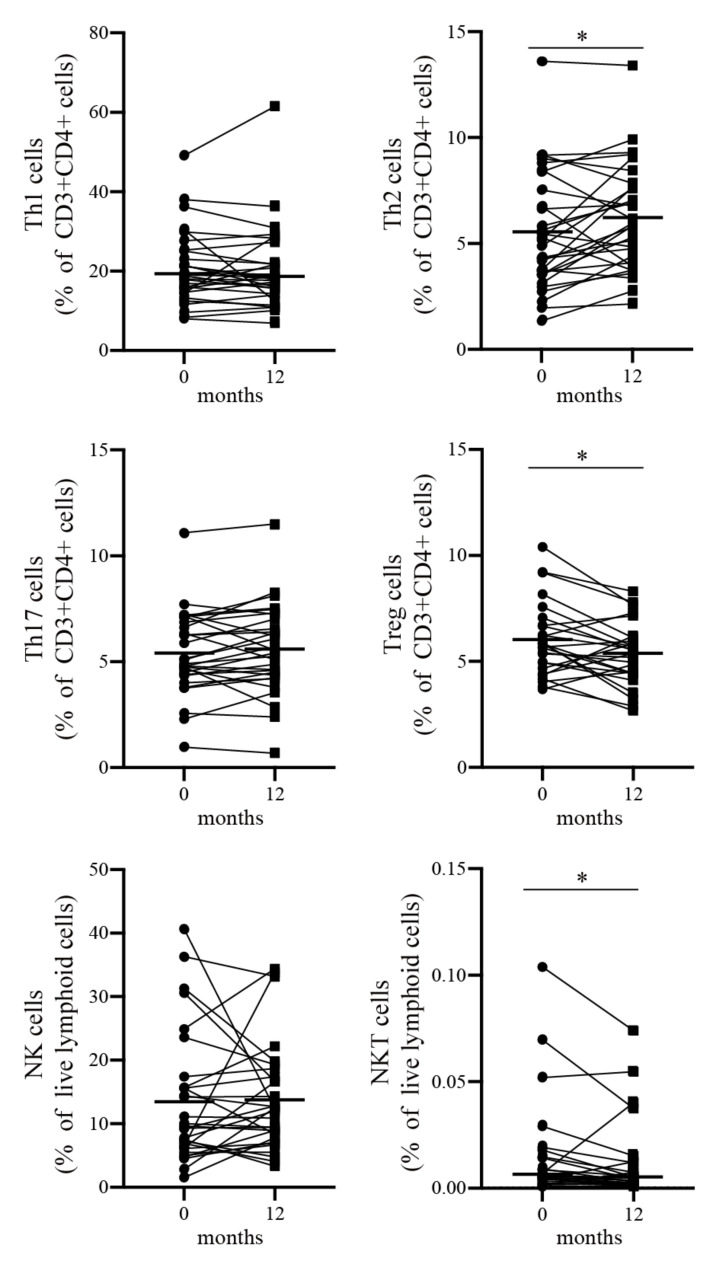
Changes in immune cell profile from baseline to 12 months after treatment with benralizumab. The frequencies of circulating Th1 cells, Th2 cells, Th17 cells, Tregs, NK cells, and NKT cells are plotted. Abbreviations: NK, natural killer; NKT, natural killer T; Th, helper T; Treg, regulatory T. Bars indicate median values. * *p* < 0.05.

Next, we examined the frequency of PBMCs in peripheral bloods using flow cytometry. The gating strategy for the PBMCs is shown in Supplementary Figure S1. The frequencies of Th cells, ILCs, and MAIT cells were demonstrated by their ratios to CD3^+^ and CD4^+^ cells, Lin^−^ CD127^+^ and CD161^+^ cells, and CD3^+^ cells, respectively. The frequencies of NK cells, NKT cells, and γδT cells were also shown by their ratios to lymphocytes. Flow cytometric analysis of peripheral blood showed that 1 year of benralizumab treatment significantly increased the frequencies of Th2 cells and significantly decreased the frequencies of Tregs and NKT cells (Table 3 and Figure 2). These findings suggest that the 1-year treatment regimen with benralizumab for patients with severe asthma reduces peripheral blood eosinophils, increases Th2 cells and serum eotaxin-1 levels, and transiently increases serum IL-5 levels.

Because these changes in each parameter after 1 year of benralizumab treatment may have been influenced by omalizumab and mepolizumab used before benralizumab treatment, we divided, into two groups, 17 patients previously treated with biologics (14 patients with mepolizumab, 3 patients treated with omalizumab) and 13 patients who were not previously treated with biologics. ACT score and FEV1% were significantly improved only in patients without previous use of biologics, and increased Th2 cells and decreased Tregs and NKT cells were significant only in patients with previous use of biologics (Supplemental Table S2). These findings showed a similar tendency in each other group, suggesting the possibility that the decrease in the number of patients due to the division had an effect. On the other hand, due to the splitting, in patients without previous use of biologics, a significant increase in serum IL-5 (although the number of patients who could be measured was small) and a significant decrease in circulating ILC1 were observed (Supplemental Table S2). These findings, at least, suggested that decreased peripheral blood eosinophils, increased Th2 cells, and increased serum eotaxin-1 levels may not be affected by previous use of biologics.

### 3.3. Parameters for Predicting the Effectiveness of Benralizumab in Patients with Severe Asthma

**Figure 3:**
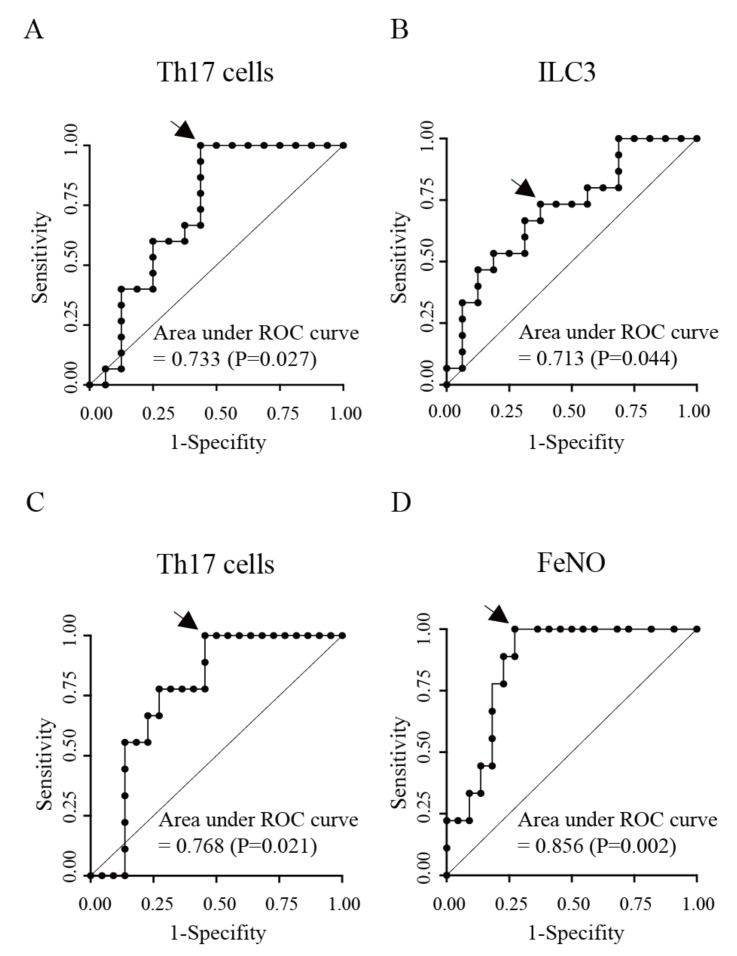
ROC curve for predicting responders (**A**,**B**) and patients achieving super-responders (**C**,**D**) to benralizumab treatment in patients with severe asthma. The cut-off values for frequency of Th17 cells of 4.57% Th cells (**A**) (sensitivity, 100%; specificity, 56.3%) and ILC3 of 11.45% ILCs (**B**) (sensitivity, 73.3%; specificity, 62.5%) to discriminate responders from non-responders are indicated with arrows. The cut-off value for FeNO levels of 44.0 ppb (**C**) (sensitivity, 100%; specificity, 72.7%) and the frequency of Th17 cells of 4.77% Th cells (**D**) (sensitivity, 100%; specificity, 54.6%) to discriminate super-responders from non-super-responders are indicated with arrows.

We then divided the 31 patients into two subgroups according to their response to benralizumab treatment (Table 4). The number of responders was 15 (48%) and they had a significantly lower average BMI and frequency of ILC3 in peripheral blood, as well as a higher mean frequency of Th17 cells before benralizumab treatment than non-responders (Table 4). Non-responders had more cases of atopic dermatitis than responders (Table 4). ROC curve analysis was used to determine the optimal cut-off values of the frequency of Th17 cells, ILC3, and BMI to discriminate responders from non-responders, with observed areas under the curve of 0.733 (*p* = 0.027), 0.713 (*p* = 0.044), and 0.704 (*p* = 0.053), respectively (Figure 3A). The frequencies of Th17 cells of 4.57% Th cells (sensitivity, 100%; specificity, 56.3%) and ILC3 of 11.45% ILCs (sensitivity, 73.3%; specificity, 62.5%) were the best cut-off values for the optimal potential effectiveness of benralizumab treatment using the Youden index [29].

Nine patients (29%) with super-responders showed the highest FeNO levels, frequency of Th17 cells in peripheral blood, and number of asthma exacerbations and unscheduled visits for worsening asthma, and lowest %FEV1, %PEFR, and %MMF before benralizumab treatment (Table 4). The areas under the ROC curves of FeNO levels and the frequency of Th17 cells were 0.856 (*p* = 0.002) and 0.768 (*p* = 0.021), respectively (Figure 3B). FeNO levels of 44.0 ppb (sensitivity, 100%; specificity, 72.7%) and Th17 cell frequencies of 4.77% Th cells (sensitivity, 100%; specificity, 54.6%) were the best cutoffs for predicting super-responders to benralizumab treatment. Therefore, we postulated that high FeNO levels and high frequencies of Th17 cells before treatment could be used as biomarkers for predicting the effectiveness of benralizumab in the treatment of patients with severe asthma.

Because this study included 18 patients with previous use of biologics, a similar analysis was performed on 13 patients without previous use of biologics. The number of responders and super-responders was 8 (62%) and 5 (38%), respectively. Responders had a significantly older age at asthma onset and no cases of atopic dermatitis than non-responders (Supplemental Table S3). Responders and super-responders showed a significantly higher frequency of Th17 cells in peripheral blood than non-responders (Supplemental Table S3). Super-responders had significantly higher FeNO levels, peripheral blood eosinophils and basophils, and serum total IgE levels (Supplemental Table S3). These findings suggested that our results of biomarkers may be less modulated by previous use of biologics.

### 3.4. Association between Type 2 Biomarkers and Non-Type 2 Biomarker Levels with Subject Characteristics in Patients with Severe Asthma

Although Th17 cells do not play a central role in type 2 inflammation, a high frequency of Th17 cells was a candidate for predicting the effects of benralizumab-targeting on eosinophils in this study. Thus, we investigated whether type 2 biomarkers, including FeNO, and non-type 2 biomarker levels, including Th17 cells and ILC3, were associated with clinical parameters of asthma. FeNO levels were positively correlated with serum total IgE levels (r = 0.498, *p* = 0.004) and serum periostin levels (r = 0.515, *p* = 0.003), but were negatively correlated with the number of hospitalizations (r = −0.476, *p* = 0.008), %FEV1 (r = −0.406, *p* = 0.023), FEV1% (r = −0.532, *p* = 0.002), MMF (r = −0.481, *p* = 0.006), and %MMF (r = −0.561, *p* = 0.001) (Table 5). The frequency of Th17 cells was positively correlated with the number of unscheduled visits for worsening asthma (r = 0.416, *p* = 0.020) and the frequency of Th2 cells (r = 0.686, *p* < 0.001), and was negatively correlated with serum IFN-γ levels (r = −0.615, *p* = 0.037) (Table 5). The frequency of ILC3 was negatively correlated with the frequency of ILC1 (r = −0.458, *p* = 0.010) (Table 5). Peripheral blood eosinophil counts were positively correlated with basophil counts (r = 0.743, *p* < 0.001), lymphocyte counts (r = 0.377, *p* = 0.037), serum periostin levels (r = 0.422, *p* = 0.018), serum IFN-γ levels (r = 0.615, *p* = 0.037), and serum MIP-1β levels (r = 0.438, *p* = 0.014), and negatively correlated with the frequency of neutrophils (r = −0.445, *p* = 0.012) and serum eotaxin-1 levels (r = −0.375, *p* = 0.038) (Table 5). Serum periostin levels were positively correlated with FeNO levels, eosinophil counts, basophil counts (r = 0.493, *p* = 0.005), and serum MIP-1β levels (r = 0.373, *p* = 0.039), and negatively correlated with FEV_1_% (r = −0.500, *p* = 0.004), MMF (r = −0.459, *p* = 0.009), %MMF (r = −0.501, *p* = 0.004), and the frequency of neutrophils (r = −0.394, *p* = 0.028) (Table 5). These findings suggest that Th17 cells and type 2 biomarkers are not correlated; however, the positive correlation between Th17 and Th2 cells suggested that Th17 cells may be involved in Th2 inflammation.

## 4. Discussion

One year of benralizumab treatment in patients with severe asthma improved their ACT scores and FEV1% values, and reduced the number of asthma exacerbations as well as unscheduled hospital visits. Benralizumab treatment also decreased peripheral blood eosinophil and basophil counts, and increased serum eotaxin-1 levels over the 1-year period and transient serum IL-5 levels up to 4 months. To our knowledge, this is the first study to show that benralizumab treatment increases circulating Th2 cells and decreases circulating Tregs after 1 year, and that the high frequency of circulating Th17 cells and high FeNO levels might predict the real-world response of benralizumab treatment in patients with severe asthma.

Similar to this real-world study, previous reports have shown that benralizumab treatment suppresses asthma symptoms and exacerbations of asthma for a year and improves airflow limitation, including FEV_1_ [9,10,11]. The decrease in basophils and eosinophils with benralizumab treatment has already been explained by the expression of IL-5Rα in basophils [30]. In the aforementioned study, peripheral blood eosinophils were absent in all patients after benralizumab treatment, while basophils were still present, even though both eosinophils and basophils expressed IL-5Rα. However, in all nine super-responders herein, peripheral blood basophils were absent after 1 year of benralizumab treatment. Elevated serum eotaxin-1 levels, a transient elevation of serum IL-5 levels, and increased peripheral blood Th2 cells were assumed to be the result of feedback due to peripheral blood eosinophil depletion. Although the involvement of IL-4 was suspected, which can induce Th2 cells and eotaxin production, serum IL-4 and IL-13 were undetectable in this study, and the feedback mechanism of how depleted peripheral blood eosinophils increased Th2 cells remains unclear. Regarding eotaxin-1, similar findings have been suggested in two previous reports that showed an increase in serum levels of eotaxin-1 and eotaxin-2 in patients with asthma following benralizumab (100 mg or 200 mg) treatment for 8 weeks or benralizumab (200 mg) treatment for 52 weeks [31,32]. The decrease in peripheral blood Tregs and NKT cells was thought to be due to the stabilization of airway inflammation during benralizumab treatment, which reduced the need for Tregs to suppress inflammation, and which might reduce CD1d-expressed dendritic cells that activate NKT cells, respectively. The findings in this study at least suggest that Tregs may not be involved in the effectiveness of benralizumab treatment. A previous real-world study in Italy showed that the peripheral percentages of NKT-like cells significantly decreased in 20 patients after 6 months of mepolizumab treatment, but not in 8 patients after benralizumab treatment [33]. This difference from our study may be attributed to the different numbers between 8 and 31 patients with benralizumab treatment. However, further case accumulation and further studies are required to confirm these assumptions.

When responders to benralizumab treatment were defined as meeting 2 of the 3 criteria, which were the improvement in ACT score, FEV_1_, and the number of exacerbations, and super-responders were defined as meeting 3 of the 3 above criteria, a high frequency of Th17 cells and low frequency of ILC3 before benralizumab treatment in responders and high FeNO levels and high frequency of Th17 cells in super-responders could be biomarkers for predicting the effectiveness after 1 year of benralizumab treatment in patients with severe asthma. Eighteen patients, more than half of whom participated in the study, had received biologics as pretreatment, which is one of the limitations of this study. Indeed, super-responders in biologics-naïve patients had high levels of type 2 inflammatory markers including higher peripheral blood eosinophil and basophil counts, serum total IgE levels, and FeNO levels, similar to previous reports. However, it was suggested that the high frequency of circulating Th17 cells before benralizumab treatment in responders and high FeNO levels and a high frequency of circulating Th17 cells in super-responders may be biomarkers for predicting the effectiveness after 1 year of benralizumab treatment in the 13 patients without biologics as pretreatment, as in the whole population of this study. A retrospective analysis of the real-world setting in the United Kingdom (UK) defined either a 50% or greater reduction in asthma exacerbation rate or a 50% reduction in OCS dose as a benralizumab responder, responders were 112 of 130 patients (86%), and the FeNO value of the responder was significantly higher than that of the non-responders, suggesting that FeNO predicts the responder [14]. Additionally, Watanabe et al. reported that benralizumab responders were 16 of 21 patients (76%) after the 24-week treatment with benralizumab, and that baseline peripheral blood eosinophil counts of 100/μL and FeNO levels of 40 ppb were the best cutoffs for predicting responders for benralizumab treatment [34]. Taken together, our results suggested that FeNO is an emerging candidate for effect prediction biomarkers other than eosinophils at least. On the other hand, parameters using flow cytometry such as the high frequency of circulating Th17 cells are not available as clinical biomarkers in practice.

Although peripheral blood Th17 cells are thought to play a central role in non-type 2 inflammation, they have been shown to up-regulate Th2-cell-mediated eosinophilic airway inflammation in a mouse model [35]. Furthermore, IL-4- and IL-17-producing Th2/Th17 cells have been reported to be associated with the co-expression of GATA-binding protein 3 and retinoic acid receptor-related orphan receptor γt, which may be carriers of type 2 inflammation [36,37]. Therefore, it is speculated that high FeNO and Th17 levels as biomarkers probably reflect type 2 inflammation. In this study, eosinophils did not function as biomarkers for predicting the effectiveness of benralizumab treatment, because many of the enrolled patients might have had low peripheral blood eosinophils due to previous treatment with OCS or biologics [38,39]. Although previous reports have shown that eosinophils are useful biomarkers, there are a certain number of asthma patients who respond to benralizumab even with low peripheral blood eosinophil counts [13,14]. Nonetheless, further studies are needed to investigate whether Th17 can be used as a type 2 biomarker to predict the therapeutic effectiveness of benralizumab in patients with severe asthma because the types of cytokines produced by peripheral blood Th17 cells analyzed in this study were unclear.

Furthermore, non-responders had a higher frequency of ILC3 and higher BMI than responders. ILC3 has been shown to be associated with obesity and asthma in a mouse model [40,41]. In addition, it has been shown that CD69^+^ ILC3 in peripheral blood correlates with BMI in patients with asthma [26]. These findings suggest that benralizumab is less effective for the treatment of patients who are obese and have asthma. There were also more patients with atopic dermatitis in non-responders than responders to benralizumab, suggesting that benralizumab is less effective for the treatment of asthmatics with atopic dermatitis.

In this study, two patients were suspected of having anaphylaxis and one had anaphylaxis as adverse events; however, the patient with anaphylaxis improved immediately and was switched from mepolizumab treatment. Nonetheless, after anaphylaxis due to benralizumab, the patient developed anaphylaxis after being re-initiated on mepolizumab [42]. It is, therefore, necessary to keep in mind the risk of anaphylaxis from the use of biologics.

Although peripheral blood eosinophils were depleted to zero in all patients 4 months after benralizumab treatment in this study, eosinophils were subsequently elevated in two patients (6.5%). One patient had worsening asthma during the 1 year of observation, and the other had worsening asthma after the 1 year of benralizumab treatment. Drug-neutralizing antibodies to benralizumab may have developed in these patients; however, these antibodies were not characterized. A real-world study in the UK also demonstrated the development of detectable blood eosinophil counts in keeping with the presumed development of drug-neutralizing antibodies in five patients (3.8%) [14]. Although anti-drug antibodies could work without impacting the treatment efficacy, especially if they are not neutralizing, the characterization of drug-neutralizing antibodies is an issue that needs to be considered in the future.

Finally, the limitation of this study was that it was a single-center, single-arm, open-label, observational study with a small sample size. In particular, the small sample size was one of the important limitations, as it can affect the numerical values of various parameters. Moreover, the components in the expert consensus-based criteria of the clinical responders and super-responders in this study have not yet had a consensus, did not remain completely consistent with previous reports, and will likely evolve and change over time. Super-responders had a lower FEV_1_ compared to the other patients, suggesting the possibility that patients with a lower FEV_1_ and more likely to be evaluated as super-responders were a limitation of this study. This study in a real-world setting, unlike a phase III clinical trial conducted by a pharmaceutical company, included patients who had asthma symptoms that could not be controlled with existing treatments, but some patients had no exacerbation before benralizumab treatment. One of the limitations of this study is that even such patients were included in the responders if they had no asthma exacerbations during the 1 year of benralizumab treatment. Furthermore, although patients with low eosinophil count immediately before benralizumab treatment were also included in this study, all patients had at least a maximum value of eosinophil counts of 150 or more during all outpatient visits. However, all patients in this study underwent extensive assessment, including treatment options other than biologics, before initiation of benralizumab. Therefore, we feel confident that the clinical improvements observed following the addition of benralizumab to the regimen of patients with uncontrolled severe asthma reflects the effectiveness of benralizumab and does not reflect any optimization of previous background therapy. This study also showed some emerging candidates for effect prediction biomarkers for benralizumab treatment, but the identification of prognostic biomarkers needs to be confirmed in further studies for validation.

## 5. Conclusions

This study showed that benralizumab treatment increased ACT scores, FEV_1_% levels, peripheral blood Th2 cells, and eotaxin-1 levels over 1 year, as well as transiently increased IL-5 levels up to 4 months, and decreased the number of asthma exacerbations, unscheduled visits, peripheral blood eosinophils, basophils, and Tregs in patients with uncontrolled severe asthma. We have provided the first report in a real-world setting that showed that 1 year of benralizumab treatment increased circulating Th2 cells and decreased circulating Tregs, and that circulating Th17 cells, ILC3, and FeNO levels might predict the effectiveness of benralizumab in the treatment of patients with severe asthma. Nevertheless, additional studies are needed to investigate whether these parameters have a broader purpose for use in the pathophysiology and treatment or management of asthma.

## Figures and Tables

**Table 1:** Baseline characteristics of the study population.

Parameter	Value (*n* = 31)
Sex (M/F), *n* (%)	9 (29%)/22 (71%)
Age (y)	54.3 ± 13.5
Age at asthma onset (y)	35.6 ± 18.6
Duration of asthma (y)	16 (8–26)
BMI (kg/m^2^)	24.2 ± 4.8
Smoking history (never/ex), *n* (%)	22 (71%)/9 (29%)
AERD, *n* (%)	5 (16.1%)
Atopic dermatitis, *n* (%)	8 (25.8%)
Allergic rhinitis, *n* (%)	25 (80.6%)
Chronic sinusitis, *n* (%)	20 (64.5%)
ABPA, *n* (%)	1 (3.2%)
Daily dose of ICS (FP equivalent dose, µg)	1000 (1000–1000)
Oral corticosteroid treatment, *n* (%)	3 (9.7%)
Previous omalizumab treatment, *n* (%)	4 (12.9%)
Previous mepolizumab treatment, *n* (%)	14 (45.2%)
Asthma exacerbations (/year)	3 (1–4)
Unscheduled visits (/year)	1 (0–2)
Hospitalizations (/year)	0 (0–1)
ACT score points	16.8 ± 5.6

For normally distributed data, number quoted is mean ± SD. For nonparametric variables, number quoted is median (interquartile range).

**Table 2:** Baseline pulmonary function and laboratory findings of the study population.

Parameter	Value (*n* = 31)
FeNO (ppb)	43 (15–74)
FVC (L)	2.9 ± 0.6
%FVC (predicted, %)	97.1 ± 14.8
FEV_1_ (L)	2.1 ± 0.7
%FEV_1_ (predicted, %)	83.2 ± 24.7
FEV_1_% (%)	70.8 ± 17.6
PEFR (L/s)	6.5 ± 2.1
%PEFR (predicted, %)	96.5 ± 27.5
MMF (L/s)	1.9 ± 1.3
%MMF (predicted, %)	58.7 ± 38.8
Neutrophils (%)	60.8 ± 8.8
Neutrophils (×10^2^ cells/μL)	32.5 (27.4–44.6)
Eosinophils (%)	1.6 (0.6–6.4)
Eosinophils (cells/μL)	80.0 (32.0–313.0)
Basophils (%)	0.6 ± 0.4
Basophils (cells/μL)	22.0 (10.6–49.7)
Lymphocytes (%)	28.0 ± 7.0
Lymphocytes (×10^2^ cells/μL)	16.5 ± 3.9
Monocytes (%)	6.3 ± 1.8
Monocytes (cells/μL)	383.1 ± 140.4
Total IgE (IU/mL)	87.0 (3.0–453.0)
Periostin (ng/mL)	74.0 (58.0–100.0)
Tenascin-C (ng/mL)	34.9 (28.1–56.5)
Eotaxin-1 (pg/mL)	124.3 ± 57.9
IFN-γ (pg/mL), *n* = 12	6.9 (3.7–17.7)
IL-5 (pg/mL), *n* = 21	4.4 (1.6–14.7)
IL-17 (pg/mL), *n* = 11	2.5 (1.3–3.4)
MCP-1 (pg/mL)	592.1 ± 212.4
MIP-1β (pg/mL)	55.4 (41.8–81.9)
RANTES (ng/mL), *n* = 30	29.1 (22.8–37.9)
Th1 cells (% of Th cells, %)	19.0 (14.7–25.1)
Th2 cells (% of Th cells, %)	5.7 ± 2.9
Th17 cells (% of Th cells, %)	5.4 ± 1.9
Treg cells (% of Th cells, %), *n* = 27	5.9 ± 1.8
ILC1 (% of ILC cells, %)	64.0 (56.6–72.9)
ILC2 (% of ILC cells, %)	23.6 (14.2–28.1)
ILC3 (% of ILC cells, %)	10.7 (8.3–17.3)
NK cells (% of lymphoid cells, %)	9.7 (6.2–17.4)
γδT cells (% of lymphoid cells, %)	2.3 (1.9–4.2)
NKT (% of lymphoid cells, ×10^−3^%), *n* = 29	5.3 (2.3–14.5)
MAIT cells (% of CD3^+^ cells, %), *n* = 30	2.0 (1.1–2.9)

For normally distributed data, number quoted is mean ± SD. For nonparametric variables, number quoted is median (interquartile range).

**Table 3:** Baseline characteristics of the study population and kinetics of parameters in patients treated with benralizumab.

Parameter	Baseline (*n* = 30)	1 Year after (*n* = 30)	*p* Value
Asthma exacerbations (/year)	3.0 (1.0–4.0)	1.0 (0.0–2.0)	0.010 *
Unscheduled visits (/year)	1.0 (0.0–2.0)	0.0 (0.0–1.0)	0.030 *
Hospitalizations (/year)	0.0 (0.0–1.0)	0.0 (0.0–0.0)	0.063
ACT score points	17.0 ± 5.7	21.0 (18.8–25.0)	0.001 *
FeNO (ppb)	44.0 (15.0–79.0)	43.0 (19.0–72.3)	0.287
FVC (L)	2.9 ± 0.6	3.0 ± 0.7	0.936
%FVC (predicted, %)	97.6 ± 14.8	98.4 ± 15.4	0.689
FEV_1_ (L)	2.1 ± 0.7	2.2 ± 0.7	0.141
%FEV_1_ (predicted, %)	83.1 ± 25.1	87.5 ± 23.5	0.034 *
FEV_1_% (%)	70.3 ± 17.7	79.1 (65.7–84.5)	0.001 *
PEFR (L/s)	6.6 ± 2.1	6.8 ± 2.1	0.358
%PEFR (predicted, %)	97.4 ± 27.5	100.5 ± 23.7	0.237
MMF (L/s)	1.9 ± 1.3	2.0 ± 1.3	0.106
%MMF (predicted, %)	58.2 ± 39.3	64.1 ± 35.7	0.072
Neutrophils (%)	60.9 ± 8.9	62.9 ± 8.8	0.197
Neutrophils (×10^2^ cells/μL)	33.0 (27.4–44.7)	33.3 (27.3–40.3)	0.952
Eosinophils (%)	1.4 (0.6–5.9)	0.0 (0.0–0.0)	<0.001 *
Eosinophils (cells/μL)	75.0 (31.8–301.8)	0.0 (0.0–0.0)	<0.001 *
Basophils (%)	0.6 ± 0.4	0.0 (0.0–0.2)	<0.001 *
Basophils (cells/μL)	22.0 (0.0–129.8)	0.0 (0.0–50.6)	<0.001 *
Lymphocytes (%)	28.1 ± 7.1	30.0 ± 7.8	0.132
Lymphocytes (×10^2^ cells/μL)	16.6 ± 3.9	16.5 ± 5.0	0.937
Monocytes (%)	6.0 ± 2.1	5.7 (4.5–7.2)	0.643
Monocytes (cells/μL)	381.5 ± 142.5	309.8 (246.5–388.7)	0.092
Total IgE (IU/mL), *n* = 29	87.0 (32.5–387.0)	119.0 (32.5–282.5)	0.960
Periostin (ng/mL)	78.5 (57.0–101.8)	79.5 (56.5–138.0)	0.171
Tenascin-C (ng/mL)	35.9 (28.7–57.9)	39.1 ± 18.4	0.371
Eotaxin-1 (pg/mL)	124.5 ± 58.9	227.0 (138.1–291.4)	<0.001 *
IFN-γ (pg/mL), *n* = 9	6.4 (3.7–28.7)	12.4 (2.9–22.9)	0.820
IL-5 (pg/mL), *n* = 20	4.9 (1.6–14.8)	7.8 (3.3–17.5)	0.674
IL-17 (pg/mL), *n* = 8	2.6 (1.4–8.7)	2.5 (1.7–4.6)	0.729
MCP-1 (pg/mL)	604.5 ± 204.3	531.1 (393.5–670.4)	0.318
MIP-1β (pg/mL)	54.7 (41.4–82.8)	69.0 ± 27.2	0.084
RANTES (ng/mL), *n* = 29	26.7 (22.3–38.5)	26.6 (19.2–33.6)	0.468
Th1 cells (% of Th cells, %)	18.9 (14.6–25.2)	18.2 (14.2–23.3)	0.526
Th2 cells (% of Th cells, %)	5.5 ± 2.8	6.2 ± 2.5	0.030 *
Th17 cells (% of Th cells, %)	5.4 ± 1.9	5.6 ± 2.1	0.258
Treg cells (% of Th cells, %), *n* = 26	6.0 ± 1.8	5.4 ± 1.5	0.016 *
ILC1 (% of ILC cells, %)	64.2 ± 12.9	61.9 ± 13.7	0.532
ILC2 (% of ILC cells, %)	23.0 ± 11.6	25.5 ± 13.2	0.224
ILC3 (% of ILC cells, %)	10.3 (8.2–17.4)	12.6 ± 9.0	0.964
NK cells (% of lymphoid cells, %)	9.5 (6.2–16.1)	12.2 (7.6–17.7)	0.670
γδT cells (% of lymphoid cells, %)	2.4 (1.9–4.2)	2.7 (1.7–3.6)	0.652
NKT (% of lymphoid cells, ×10^−3^%), *n* = 27	5.4 (3.3–14.7)	4.2 (2.0–12.0)	0.011 *
MAIT cells (% of CD3^+^ cells, %), *n* = 29	2.0 (1.0–3.0)	2.1 ± 1.3	0.658

For normally distributed data, number quoted is mean ± SD. For nonparametric variables, number quoted is median (interquartile range). * *p* < 0.05.

**Table 4:** Responder analysis of benralizumab treatment.

Parameter	Responder (*n* = 15)	Non-Responder (*n* = 16)	*p* Value	Super-Responder (*n* = 9)	Others (*n* = 22)	*p* Value
Sex (M/F), *n* (%)	5 (33.3%)/10 (66.7%)	4 (25.0%)/12 (75.0%)	0.704	5 (55.6%)/4 (44.4%)	4 (18.2%)/18(81.8%)	0.077
Age (y)	55.1 ± 9.8	53.4 ± 16.6	0.730	54.0 ± 9.9	54.4 ± 15.0	0.938
Age at asthma onset (y)	41.3 ± 15.6	30.2 ± 20.0	0.094	38.6 ± 14.8	34.4 ± 20.2	0.529
Duration of asthma (y)	13.8 ± 11.5	17.5 (8.0–60.0)	0.058	15.4 ± 11.0	16.5 (8.8–26.5)	0.571
BMI (kg/m^2^)	22.5 ± 4.4	25.9 ± 4.7	0.049 *	23.4 ± 4.5	24.6 ± 4.9	0.514
Smoking history (never/ex), *n* (%)	10 (66.7%)/5 (33.3%)	12 (75.0%)/4 (25.0%)	0.704	6 (66.7%)/3 (33.3%)	16 (72.7%)/6 (27.3%)	>0.999
AERD, *n* (%)	3 (20.0%)	2 (12.5%)	0.654	1 (11.1%)	4 (18.2%)	>0.999
Atopic dermatitis, *n* (%)	1 (6.7%)	7 (43.8%)	0.004 *	0 (0.0%)	8 (36.4%)	0.068
Allergic rhinitis, *n* (%)	12 (80.0%)	13 (81.3%)	>0.999	8 (88.9%)	17 (77.3%)	0.642
Chronic sinusitis, *n* (%)	10 (66.7%)	10 (62.5%)	>0.999	6 (66.7%)	14 (63.6%)	>0.999
ABPA, *n* (%)	0 (0.0%)	1 (6.3%)	>0.999	0 (0.0%)	1 (4.5%)	>0.999
Daily dose of ICS (FP equivalent dose, µg)	873.3 ± 246.3	1000.0 (1000.0–1000.0)	0.119	1000.0 (800.0–1000.0)	1000.0 (1000.0–1000.0)	0.570
Oral corticosteroid treatment, *n* (%)	0 (0.0%)	3 (18.8%)	0.238	0 (0.0%)	3 (13.6%)	0.538
Previous omalizumab treatment, *n* (%)	1 (6.7%)	3 (18.8%)	0.600	0 (0.0%)	4 (18.2%)	0.295
Previous mepolizumab treatment, *n* (%)	6 (40.0%)	8 (50.0%)	0.722	4 (44.4%)	10 (45.5%)	>0.999
Asthma exacerbations (/year)	3.0 (0.0–5.0)	2.4 ± 1.5	0.685	4.0 (2.0–11.5)	2.0 ± 1.6	0.021 *
Unscheduled visits (/year)	1.0 (0.0–3.0)	0.8 ± 1.1	0.158	2.0 (0.5–4.5)	0.8 ± 1.1	0.043 *
Hospitalizations (/year)	0.0 (0.0–0.3)	0.0 (0.0–1.0)	0.587	0.0 (0.0–0.0)	0.0 (0.0–1.0)	0.362
ACT score points	16.0 ± 6.7	17.6 ± 4.5	0.437	15.4 ± 6.9	17.4 ± 5.1	0.454
FeNO (ppb)	49.0 (17.0–102.0)	20.5 (11.3–48.3)	0.091	66.0 (48.0–115.0)	18.0 (10.0–46.8)	0.001 *
FVC (L)	2.9 ± 0.7	2.9 ± 0.6	0.824	3.0 ± 0.8	2.9 ± 0.6	0.849
%FVC (predicted, %)	97.3 ± 18.1	96.9 ± 11.5	0.950	88.5 ± 17.2	100.6 ± 12.5	0.081
FEV_1_ (L)	2.0 ± 0.7	2.2 ± 0.7	0.435	1.9 ± 0.8	2.2 ± 0.7	0.302
%FEV_1_ (predicted, %)	79.0 ± 27.1	87.1 ± 22.3	0.373	66.0 ± 24.3	90.2 ± 21.6	0.021 *
FEV_1_% (%)	66.6 ± 17.6	82.9 (66.8–85.3)	0.132	61.6 ± 17.1	82.0 (62.3–85.2)	0.056
PEFR (L/s)	6.2 ± 2.3	6.9 ± 1.9	0.371	6.1 ± 2.6	6.7 ± 1.9	0.517
%PEFR (predicted, %)	90.2 ± 28.1	102.5 ± 26.4	0.222	78.1 ± 26.6	104.1 ± 24.5	0.024 *
MMF (L/s)	1.5 ± 1.2	2.2 ± 1.4	0.161	0.6 (0.6–1.7)	2.2 ± 1.4	0.101
%MMF (predicted, %)	49.2 ± 40.1	67.5 ± 36.5	0.196	32.9 ± 22.9	69.2 ± 39.3	0.004 *
Neutrophils (%)	61.2 ± 10.5	60.4 ± 7.2	0.814	59.4 ± 10.3	61.3 ± 8.3	0.629
Neutrophils (×10^2^ cells/μL)	34.1 (30.1–45.1)	30.7 (26.5–43.6)	0.626	32.5 (27.9–40.7)	33.1 (27.4–46.2)	0.881
Eosinophils (%)	1.1 (0.6–8.1)	2.9 ± 3.1	0.605	5.7 (0.6–9.2)	1.4 (0.6–4.3)	0.361
Eosinophils (cells/μL)	61.0 (41.0–583.0)	86.0 (29.5–228.0)	0.626	188.1 (44.0–693.0)	75.0 (30.5–301.8)	0.403
Basophils (%)	0.7 ± 0.5	0.5 ± 0.3	0.262	0.7 ± 0.5	0.5 ± 0.4	0.192
Basophils (cells/μL)	39.8 ± 31.2	21.5 (10.6–42.8)	0.488	45.2 ± 34.1	20.7 (10.6–44.4)	0.207
Lymphocytes (%)	26.2 ± 6.1	29.8 ± 7.5	0.150	26.5 ± 6.3	28.7 ± 7.3	0.412
Lymphocytes (×10^2^ cells/μL)	15.8 ± 4.6	17.1 (14.4–19.9)	0.264	15.6 ± 4.7	16.9 ± 3.5	0.459
Monocytes (%)	6.2 ± 2.2	5.9 (5.4–7.4)	0.565	7.2 ± 2.1	6.0 ± 1.6	0.158
Monocytes (cells/μL)	373.4 ± 139.7	392.3 ± 145.1	0.715	426.0 ± 158.6	322.3 (275.6–421.7)	0.259
Total IgE (IU/mL)	122.0 (41.0–453.0)	72.0 (31.3–800.3)	0.838	201.0 (76.5–567.0)	70.0 (24.5–480.8)	0.177
Periostin (ng/mL)	94.4 ± 48.7	68.5 (51.3–89.5)	0.313	90.0 (73.5–109.0)	68.5 (47.3–92.5)	0.121
Tenascin-C (ng/mL)	40.6 ± 17.4	37.0 (25.7–59.0)	0.869	42.6 ± 17.6	35.2 (26.7–50.6)	0.710
Eotaxin-1 (pg/mL)	118.6 ± 53.2	129.7 ± 63.2	0.602	114.6 ± 43.3	128.3 ± 63.4	0.494
IFN-γ (pg/mL)	5.0 (2.7–79.1) (*n* = 6)	7.4 (5.2–25.8) (*n* = 6)	0.394	3.4 (1.7–10.2) (*n* = 4)	7.4 (5.8–38.6) (*n* = 8)	0.073
IL-5 (pg/mL)	4.2 (2.2–10.1) (*n* = 10)	4.4 (1.1–22.2) (*n* = 11)	0.918	7.4 (3.0–10.5) (*n* = 7)	3.4 (1.5–16.7) (*n* = 14)	0.689
IL-17 (pg/mL)	3.0 (1.6–45.9) (*n* = 4)	1.8 (1.1–2.8) (*n* = 7)	0.252	2.4 (1.3–3.4) (*n* = 2)	2.6 (1.2–6.6) (*n* = 9)	0.946
MCP-1 (pg/mL)	604.4 ± 217.8	580.5 ± 213.7	0.761	634.1 ± 247.2	574.9 ± 200.3	0.535
MIP-1β (pg/mL)	68.5 ± 33.6	63.5 ± 27.1 (*n* = 15)	0.654	79.3 ± 35.3	54.0 (39.3–71.4) (*n* = 21)	0.126
RANTES (ng/mL)	33.3 ± 17.4	26.3 (24.2–36.5)	0.711	37.7 ± 18.8	25.6 (22.6–36.7)	0.322
Th1 cells (% of Th cells, %)	21.1 ± 8.6	19.0 (13.9–24.6)	0.808	18.2 ± 5.6	19.3 (14.1–26.5)	0.513
Th2 cells (% of Th cells, %)	6.0 ± 2.3	5.4 ± 3.4	0.584	6.4 ± 2.2	5.4 ± 3.1	0.322
Th17 cells (% of Th cells, %)	6.3 (4.8–7.1)	4.4 (3.8–6.1)	0.027 *	6.4 ± 1.0	4.8 (3.9–6.3)	0.020 *
Treg cells (% of Th cells, %)	6.0 ± 2.0	5.8 ± 1.7 (*n* = 12)	0.734	6.5 ± 2.0	5.6 ± 1.7 (*n* = 18)	0.300
ILC1 (% of ILC cells, %)	66.8 ± 12.5	62.3 ± 13.0	0.332	61.0 ± 10.2	65.9 ± 13.6	0.283
ILC2 (% of ILC cells, %)	23.1 ± 11.3	22.5 ± 12.0	0.891	26.9 ± 11.4	21.1 ± 11.3	0.219
ILC3 (% of ILC cells, %)	10.2 ± 4.6	15.3 ± 7.9	0.039 *	12.2 ± 4.4	13.0 ± 7.8	0.712
NK cells (% of lymphoid cells, %)	16.5 ± 11.3	10.8 ± 8.3	0.119	16.2 ± 9.3	7.6 (5.4–16.4)	0.111
γδT cells (% of lymphoid cells, %)	2.5 (2.1–4.2)	2.0 (1.4–4.1)	0.476	2.3 ± 0.9	2.4 (1.6–4.4)	0.542
NKT (% of lymphoid cells, ×10^−3^%)	5.4 (2.7–46.1) (*n* = 13)	6.9 ± 5.4 (*n* = 15)	0.440	6.1 (5.2–29.1) (n = 7)	5.1 (2.3–11.6) (*n* = 21)	0.272
MAIT cells (% of CD3^+^ cells, %)	2.1 (1.4–2.9)	2.0 ± 1.3 (*n* = 15)	0.705	1.8 ± 1.0	2.3 (1.0–3.1) (*n* = 21)	0.618

For normally distributed data, number quoted is mean ± SD. For nonparametric variables, number quoted is median (interquartile range). * *p* < 0.05.

**Table 5:** The correlation coefficients between Th17, ILC3, FeNO, eosinophils, and serum periostin levels.

Parameter	Th17 (% of Th Cells, %)		ILC3 (% of ILC Cells, %)		FeNO (ppb)		Eosinophils (Cells/μL)		Periostin (ng/mL)	
	r	*p* Value	r	*p* Value	r	*p* Value	r	*p* Value	r	*p* Value
Age (y)	0.140	0.452	0.032	0.862	0.136	0.467	−0.112	0.548	0.229	0.215
Duration of asthma (y)	0.168	0.367	0.339	0.062	−0.057	0.760	−0.071	0.706	−0.046	0.807
BMI (kg/m^2^)	−0.081	0.665	0.140	0.452	−0.153	0.411	0.216	0.243	−0.188	0.312
Asthma exacerbations (/year)	0.177	0.340	0.015	0.936	0.041	0.826	0.148	0.427	−0.106	0.572
Unscheduled visits (/year)	0.416	0.020 *	−0.113	0.545	−0.114	0.543	0.197	0.288	−0.051	0.787
Hospitalizations (/year)	0.240	0.201	−0.078	0.682	−0.476	0.008 *	−0.104	0.586	−0.251	0.180
ACT score points	−0.129	0.489	−0.084	0.654	0.183	0.323	−0.002	0.991	0.094	0.614
FeNO (ppb)	0.145	0.435	0.183	0.325	NA	NA	0.240	0.193	0.515	0.003 *
FVC (L)	−0.237	0.199	0.267	0.146	0.143	0.444	0.270	0.142	0.080	0.668
%FVC (predicted, %)	−0.112	0.548	−0.114	0.541	0.038	0.839	−0.130	0.486	0.171	0.357
FEV_1_ (L)	−0.247	0.181	0.020	0.917	−0.242	0.189	0.043	0.817	−0.208	0.261
%FEV_1_ (predicted, %)	−0.124	0.506	−0.216	0.243	−0.406	0.023 *	−0.261	0.156	−0.292	0.111
FEV_1_% (%)	−0.103	0.582	−0.163	0.381	−0.532	0.002 *	−0.203	0.273	−0.500	0.004 *
PEFR (L/s)	−0.154	0.409	0.015	0.936	−0.041	0.825	0.154	0.409	−0.042	0.822
%PEFR (predicted, %)	0.028	0.882	−0.265	0.150	−0.223	0.228	−0.214	0.248	−0.109	0.560
MMF (L/s)	−0.057	0.762	−0.114	0.542	−0.481	0.006 *	−0.127	0.496	−0.459	0.009 *
%MMF (predicted, %)	0.036	0.848	−0.189	0.309	−0.561	0.001 *	−0.257	0.162	−0.501	0.004 *
Neutrophils (%)	−0.077	0.680	0.030	0.872	−0.105	0.573	−0.445	0.012 *	−0.394	0.028 *
Neutrophils (×10^2^ cells/μL)	−0.194	0.297	−0.030	0.873	0.055	0.768	0.167	0.369	−0.138	0.459
Eosinophils (%)	0.138	0.459	0.005	0.978	0.266	0.149	0.970	<0.001*	0.457	0.010 *
Eosinophils (cells/μL)	0.109	0.558	0.018	0.925	0.240	0.193	NA	NA	0.422	0.018 *
Basophils (%)	−0.006	0.974	0.118	0.528	0.290	0.113	0.650	<0.001 *	0.461	0.009 *
Basophils (cells/μL)	0.074	0.693	0.165	0.376	0.326	0.073	0.743	<0.001 *	0.493	0.005 *
Lymphocytes (%)	0.083	0.657	0.005	0.978	−0.114	0.543	−0.134	0.473	0.197	0.287
Lymphocytes (×10^2^ cells/μL)	−0.053	0.779	0.099	0.597	0.003	0.988	0.377	0.037 *	0.206	0.266
Monocytes (%)	0.002	0.993	0.142	0.447	−0.083	0.658	0.026	0.889	−0.016	0.933
Monocytes (cells/μL)	−0.142	0.446	−0.018	0.924	−0.051	0.784	0.317	0.083	−0.092	0.621
Total IgE (IU/mL)	0.193	0.299	0.261	0.156	0.498	0.004 *	0.156	0.401	0.347	0.056
Periostin (ng/mL)	0.337	0.064	0.010	0.957	0.515	0.003 *	0.422	0.018 *	NA	NA
Tenascin-C (ng/mL)	0.036	0.848	0.086	0.647	0.232	0.210	−0.110	0.556	0.216	0.243
Eotaxin-1 (pg/mL)	0.283	0.123	−0.255	0.166	−0.063	0.735	−0.375	0.038 *	0.217	0.241
IFN-γ (pg/mL)	−0.615	0.037 *	0.028	0.939	0.042	0.904	0.615	0.037 *	0.182	0.573
IL-5 (pg/mL)	−0.022	0.924	0.245	0.284	0.408	0.066	−0.203	0.378	0.414	0.062
IL-17 (pg/mL)	−0.155	0.647	−0.296	0.374	0.087	0.799	0.141	0.678	0.059	0.866
MCP-1 (pg/mL)	−0.021	0.912	0.042	0.823	0.283	0.122	0.034	0.856	0.347	0.056
MIP-1β (pg/mL)	0.191	0.303	0.196	0.290	0.172	0.353	0.438	0.014 *	0.373	0.039 *
RANTES (pg/mL)	−0.205	0.269	0.316	0.083	0.058	0.756	−0.134	0.471	0.042	0.822
Th1 cells (% of Th cells, %)	0.104	0.576	−0.091	0.627	−0.141	0.448	−0.279	0.128	0.010	0.957
Th2 cells (% of Th cells, %)	0.686	<0.001 *	−0.327	0.073	0.117	0.530	0.214	0.247	0.191	0.304
Th17 cells (% of Th cells, %)	NA	NA	−0.290	0.113	0.145	0.435	0.109	0.558	0.337	0.064
Treg cells (% of Th cells, %)	0.091	0.652	0.311	0.115	0.266	0.180	0.018	0.929	0.144	0.473
ILC1 (% of ILC cells, %)	0.122	0.513	−0.458	0.010 *	−0.108	0.564	−0.059	0.751	−0.091	0.628
ILC2 (% of ILC cells, %)	−0.001	0.994	−0.033	0.860	−0.112	0.549	0.014	0.941	−0.006	0.976
ILC3 (% of ILC cells, %)	−0.290	0.113	NA	NA	0.183	0.325	0.018	0.925	0.010	0.957
NK cells (% of lymphoid cells, %)	0.138	0.461	−0.154	0.409	0.250	0.175	0.285	0.120	0.190	0.306
γδT cells (% of CD3^+^ cells, %)	−0.181	0.330	0.011	0.953	−0.081	0.664	0.105	0.575	0.075	0.689
NKT (% of lymphoid cells, %)	0.017	0.931	0.183	0.343	−0.171	0.375	−0.139	0.472	−0.301	0.112
MAIT cells (% of CD3^+^ cells, %)	−0.083	0.664	0.099	0.602	−0.069	0.716	−0.058	0.761	−0.327	0.078

* *p* < 0.05.

## Data Availability

Not applicable.

## Supplementary Materials

The following supporting information can be downloaded at: https://www.mdpi.com/article/10.3390/biom13030538/s1, Figure S1: The gating strategy for the PBMCs; Table S1: The detection limits of the multiplex bead array assays and ELISA; Table S2: Baseline characteristics of the study population and kinetics of parameters in patients with/without previous use of biologics; Table S3: Responder analysis of benralizumab treatment in patients without previous use of biologics.

## Abbreviations

ACT: asthma control test; ABPA, allergic bronchopulmonary aspergillosis; AERD, aspirin-exacerbated respiratory disease; FeNO, fractional exhaled nitric oxide; FP, fluticasone propionate; FVC, forced vital capacity; FEV1%, forced expiratory volume in 1 s/forced vital capacity; ICS, inhaled corticosteroid; ILC1, group 1 ILC; ILC3, group 3 ILC; MAIT, mucosal-associated invariant T; MIP, macrophage inflammatory protein; NA, not applicable; NK, natural killer; NKT, natural killer T; OCS, oral corticosteroids; RANTES, regulated on activation normal T cell expressed and secreted; TCR, T cell antigen receptor; Tregs, regulatory T cells.
