# Supplementary material for: Pretreatment Frequency of Circulating Th17 Cells and FeNO Levels Predicted the Real-World Response after 1 Year of Benralizumab Treatment in Patients with Severe Asthma
Source: Biomolecules. 2023 Mar 15;13(3):538. doi: 10.3390/biom13030538 (PMC10046637; doi:10.3390/biom13030538)

Supplemental Table S1. The detection limits of the multiplex bead array assays and ELISA

Target	Assay working range (pg/mL)	
	LLOQ	ULOQ
Periostin	0.0	2,000.0
Tenascin-C	380.0	24,000.0
Eotaxin-1	3.2	10,000.0
IFN-γ	3.2	10,000.0
IL-4	3.2	10,000.0
IL-5	3.2	10,000.0
IL-13	3.2	10,000.0
IL-17	3.2	10,000.0
MCP-1	3.2	10,000.0
MIP-1α	3.2	10,000.0
MIP-1β	3.2	10,000.0
RANTES	15.6	1,000.0

Abbreviations: IL, interleukin; IFN-γ, interferon-gamma; LLOQ, lower limit of quantitation; ULOQ, upper limit of quantitation; MIP,macrophage inflammatory protein; MCP, monocyte chemotactic protein; RANTES,regulated on activation normal T cell expressed and secreted.

Supplementary Table S2. Baseline characteristics of the study population and kinetics of parameters in patients with/without previous use of biologics.

	Patients without previous use of biologics			Patients with previous use of biologics		
	Baseline (n = 13)	1 year after (n = 13)	*p* value	Baseline (n = 17)	1 year after (n = 17)	*p* value
Asthma exacerbations (/year)	3.0 (1.0 – 4.0)	0.0 (0.0 – 2.0)	0.065	2.0 (0.5 – 3.5)	1.6 ± 1.4	0.132
Unscheduled visits (/year)	1.0 (0.0 – 3.0)	0.5 ± 0.7	0.055	0.9 ± 1.2	0.0 (0.0 – 1.0)	0.563
Hospitalizations (/year)	0.3 ± 0.5	0.0 (0.0 – 0.0)	0.250	0.0 (0.0 – 0.0)	0.0 (0.0 – 0.0)	0.500
ACT score points	16.8 ± 6.2	24.0 (18.5 – 25.0)	0.020*	17.1 ± 5.4	20.2 ± 3.7	0.057
FeNO (ppb)	43.0 (12.5 – 71.5)	32.0 (19.0 – 72.0)	0.278	54.0 ± 42.0	49.0 (21.5 – 80.0)	0.826
FVC (L)	3.2 ± 0.6	3.3 ± 0.7	0.845	2.7 ± 0.6	2.7 ± 0.5	0.886
%FVC (predicted, %)	95.9 ± 16.5	96.6 ± 17.1	0.842	98.8 ± 13.7	99.7 ± 14.4	0.725
FEV_1 (L)	2.4 ± 0.7	2.6 ± 0.7	0.243	1.8 ± 0.6	1.9 ± 0.6	0.389
%FEV_1 (predicted, %)	84.2 ± 24.1	89.8 ± 22.6	0.133	82.3 ± 26.6	85.8 ± 24.8	0.154
FEV_1% (%)	73.1 ± 15.0	77.6 ± 13.2	0.007*	68.1 ± 19.7	70.3 ± 17.4	0.091
PEFR (L/s)	7.1 ± 2.0	7.6 ± 2.1	0.187	6.2 ± 2.1	6.2 ± 1.8	0.841
%PEFR (predicted, %)	92.0 ± 23.7	98.6 ± 22.6	0.091	101.5 ± 30.1	101.9 ± 25.1	0.923
MMF (L/s)	2.2 ± 1.4	2.6 ± 1.4	0.081	1.6 ± 1.2	1.6 ± 1.1	0.773
%MMF (predicted, %)	61.4 ± 37.6	71.7 ± 33.8	0.065	55.7 ± 41.5	58.4 ± 37.0	0.520
Neutrophils (%)	61.2 ± 10.3	63.7 (57.1 – 68.4)	0.191	60.7 ± 8.0	62.1 ± 8.5	0.528
Neutrophils (×10^2cells/μL)	41.3 ± 18.4	36.9 (25.8 – 44.4)	0.735	32.5 (27.4 – 39.7)	32.7 (26.7 – 38.3)	0.818
Eosinophils (%)	4.6 (0.7 – 8.9)	0.0 (0.0 – 0.0)	< 0.001*	1.0 (0.5 – 2.9)	0.0 (0.0 – 0.0)	0.003*
Eosinophils (cells/μL)	188.1 (46.3 – 693.0)	0.0 (0.0 – 0.0)	< 0.001*	48.0 (29.5 – 200.5)	0.0 (0.0 – 0.0)	0.003*
Basophils (%)	0.7 ± 0.5	0.0 (0.0 – 0.2)	0.001*	0.5 ± 0.3	0.0 (0.0 – 0.2)	0.002*
Basophils (cells/μL)	44.4 ± 40.2	5.1 ± 7.5	0.005*	29.2 ± 21.4	0.0 (0.0 – 10.9)	< 0.001*
Lymphocytes (%)	26.3 ± 7.5	29.5 ± 8.5	0.053	29.5 ± 6.7	30.5 ± 7.5	0.617
Lymphocytes (×10^2cells/μL)	16.5 ± 4.5	16.6 ± 5.2	0.884	17.0 ± 3.6	16.5 ± 5.0	0.801
Monocytes (%)	6.0 ± 2.1	6.4 ± 1.9	0.602	6.4 ± 1.9	5.5 (4.3 – 6.7)	0.269
Monocytes (cells/μL)	397.6 ± 150.7	366.8 ± 127.7	0.361	319.0 (281.1 – 404.3)	272.0 (229.3 – 352.8)	0.132
Total IgE (IU/ mL)	87.0 (21.5 – 342.0)	83.0 (24.0 – 270.5)	0.806	80.0 (47.8 – 800.3) (n = 16)	144.5 (54.5 – 858.5) (n = 16)	> 0.999
Periostin (ng/ mL)	83.4 ± 55.7	92.5 ± 49.6	0.352	83.0 (66.0 – 105.5)	79.0 (59.5 – 122.5)	0.438
Tenascin-C (ng/mL)	29.5 (25.2 – 59.9)	29.4 (18.5 – 40.6)	0.168	43.3 ± 16.7	41.9 ± 15.3	0.699
Eotaxin-1 (pg/mL)	97.2 ± 50.3	189.3 ± 76.7	< 0.001*	145.4 ± 57.5	274.9 (149.2 – 347.4)	< 0.001*
IFN-γ (pg/mL)	7.4 (3.4 – 145.8) (n = 5)	12.4 (7.2 – 45.1) (n = 5)	0.813	5.1 (3.7 – 35.3) (n = 4)	8.1 (1.5 – 18.1) (n = 4)	0.625
IL-5 (pg/mL)	1.4 (0.9 – 8.0) (n = 6)	12.4 (5.8 – 25.0) (n = 6)	0.031*	8.0 (2.3 – 27.6) (n =14)	6.0 (2.8 – 17.0) (n = 14)	0.194
IL-17 (pg/mL)	2.6 (1.5 – 31.8) (n = 5)	2.5 (1.7 – 10.5) (n = 5)	0.729	2.6 (1.3 – 10.5) (n =3)	2.6 (0.7 – 4.6) (n =3)	0.729
MCP-1 (pg/mL)	573.2 ± 233.3	522.0 (357.2 – 620.6)	0.542	628.4 ± 182.8	591.3 ± 182.6	0.281
MIP-1β (pg/mL)	74.7 ± 35.8	79.7 ± 30.4	0.337	49.4 (40.1 – 71.4)	60.8 ± 22.0	0.459
RANTES (ng/mL)	28.2 ± 13.8 (n = 12)	28.5 (18.3 – 31.1) (n = 12)	0.791	29.1 (23.5 – 38.3)	29.1 ± 14.8	0.225
Th1 cells (% of Th cells, %)	20.7 ± 8.3	18.7 ± 7.4	0.204	18.8 (14.6 – 23.3)	18.5 (15.7 – 24.5)	0.963
Th2 cells (% of Th cells, %)	4.6 ± 2.1	5.2 ± 2.0	0.232	6.2 ± 3.1	6.9 ± 2.6	0.075
Th17 cells (% of Th cells, %)	5.0 ± 1.7	4.9 ± 1.8	0.817	5.7 ± 2.1	6.1 ± 2.2	0.037*
Treg cells (% of Th cells, %)	5.2 (4.2 – 7.0) (n = 12)	5.3 ± 1.5 (n = 12)	0.331	6.3 ± 1.6 (n = 14)	5.5 ± 1.6 (n = 14)	0.020*
ILC1 (% of ILC cells, %)	65.1 ± 15.2	56.3 ± 9.7	0.0498*	63.5 ± 11.3	66.2 ± 15.0	0.477
ILC2 (% of ILC cells, %)	23.3 ± 14.2	29.0 ± 13.1	0.102	22.7 ± 9.6	22.9 ± 13.0	0.941
ILC3 (% of ILC cells, %)	11.6 ± 6.6	14.7 ± 8.9	0.344	9.9 (8.6 – 17.6)	10.9 ± 9.0	0.424
NK cells (% of lymphoid cells, %)	13.7 ± 10.4	16.9 ± 10.7	0.252	10.0 (5.8 – 16.5)	11.3 ± 5.3	0.712
γδT cells (% of lymphoid cells, %)	2.7 ± 1.2	2.6 (2.0 – 3.1)	0.787	2.3 (1.4 – 4.4)	2.6 (1.4 – 4.3)	0.782
NKT (% of lymphoid cells, ×10^{-3}%)	7.7 ± 6.7 (n = 11)	4.8 (2.2 – 7.5) (n = 11)	0.320	5.3 (4.0 – 25.4) (n=16)	4.0 (1.9 – 14.5) (n=16)	0.013*

MAIT cells (% of $CD3^+$cells, %)	2.2 ± 1.5	1.6 ± 0.9	0.173	2.9 (1.3 – 2.5) (n=16)	1.8 ± 1.0 (n=16)	0.155

For normally distributed data, number quoted is mean ± SD.

For nonparametric variables, number quoted is median (interquartile range).

*$p < 0.05$

Supplemental Table S3. Responder analysis of benralizumab treatment in patients without previous use of biologics.

	Responder (n = 8)	Non-responder (n = 5)	*P* value	Super-Responder (n = 5)	Others (n = 8)	*p* value
Sex (M/F), n (%)	4 (50.0%)/ 4 (50.0%)	2 (40.0%)/ 3 (60.0%)	> 0.999	4 (80.0%)/ 1 (20.0%)	2 (25.0%)/ 6 (75.0%)	0.103
Age (y)	52.6 ± 11.7	42.0 (24.5 – 58.5)	0.222	53.0 (41.5 – 55.5)	47.8 ± 18.8	0.833
Age at asthma onset (y)	41.9 ± 16.3	10.0 (6.0 – 21.0)	0.002*	29.0 (25.5 – 51.5)	27.0 ± 22.8	0.237
Duration of asthma (y)	10.8 ± 10.6	20.0 (12.0 – 50.0)	0.062	7.0 (3.5 – 25.0)	20.8 ± 19.9	0.550
BMI (kg/m^2)	24.4 ± 4.6	23.3 (18.5 – 29.4)	0.943	25.2 (20.7 – 30.2)	23.5 ± 4.6	0.524
Smoking history (never/ex), n (%)	5 (62.5%)/3 (37.5%)	4 (80.0%)/1 (20.0%)	> 0.999	2 (40.0%)/ 3 (60.0%)	2 (25.0%)/ 6 (75.0%)	> 0.999
AERD, n (%)	2 (25.0%)	1 (20.0%)	> 0.999	1 (20.0%)	2 (25.0%)	> 0.999
Atopic dermatitis, n (%)	0 (0.0%)	3 (60.0%)	0.035*	0 (0.0%)	3 (37.5%)	0.231
Allergic rhinitis, n (%)	7 (87.5%)	4 (80.0%)	> 0.999	5 (100.0%)	6 (75.0%)	0.487
Chronic sinusitis, n (%)	6 (75.0%)	3 (60.0%)	> 0.999	4 (80.0%)	5 (62.5%)	> 0.999
ABPA, n (%)	0 (0.0%)	1 (20.0%)	0.385	0 (0.0%)	1 (12.5%)	> 0.999
Daily dose of ICS (FP equivalent dose, μg)	812.5 ± 241.7	1000.0 (1000.0 – 1000.0)	0.161	800.0 (600.0 – 1000.0)	1000.0 (1000.0 – 1000.0)	0.189
Oral corticosteroid treatment, n (%)	0 (0.0%)	2 (40.0%)	0.128	0 (0.0%)	2 (25.0%)	0.487
Daily dose of OCS (PSL equivalent dose, mg)	0.0 (0.0 – 0.0)	0.0 (0.0 – 5.0)	0.128	0.0 (0.0 – 0.0)	1.3 ± 2.3	0.487
Asthma exacerbations (/year)	3.5 (1.3 – 4.8)	3.0 (0.5 – 3.5)	0.411	4.0 (2.5 – 14.5)	2.0 ± 1.5	0.051
Unscheduled visits (/year)	1.5 (0.0 – 5.3)	1.0 (0.0 – 2.0)	0.549	2.0 (0.5 – 9.0)	1.0 ± 1.3	0.183
Hospitalizations (/year)	0.0 (0.0 – 1.0)	0.0 (0.0 – 1.0)	> 0.999	0.0 (0.0 – 0.0)	0.5 (0.0 – 1.0)	0.208
ACT score points	15.4 ± 7.2	20.0 (15.5 – 22.0)	0.523	14.0 (9.0 – 20.0)	20.5 (14.3 – 22.8)	0.220
FeNO (ppb)	48.0 (23.5 – 100.0)	15.0 (10.0 – 29.0)	0.052	94.0 (48.0 – 201.0)	20.0 ± 14.6	0.002*
FVC (L)	3.2 ± 0.7	3.3 (2.9 – 3.6)	0.697	3.4 (3.0 – 3.9)	3.1 ± 0.6	0.594
%FVC (predicted, %)	97.8 ± 19.7	92.7 (83.6 – 102.2)	0.622	81.6 (72.2 – 115.8)	98.7 ± 12.5	0.524
FEV_1 (L)	2.2 ± 0.65	2.9 (2.0 – 3.2)	0.302	2.5 (1.7 – 2.9)	2.5 ± 0.8	0.547
%FEV_1 (predicted, %)	79.0 ± 27.1	92.9 (66.4 – 105.0)	> 0.999	78.7 (47.2 – 96.9)	91.0 ± 21.9	0.284
FEV_1% (%)	69.4 ± 13.3	83.7 (65.7 – 90.1)	0.284	64.2 (55.4 – 80.0)	76.9 ± 15.7	0.284
PEFR (L/s)	7.1 ± 2.3	7.7 (5.4 – 8.9)	0.979	6.6 (5.8 – 9.7)	6.9 ± 2.1	0.412
%PEFR (predicted, %)	93.8 ± 24.9	93.3 (65.3 – 110.8)	> 0.999	100.8 (61.2 – 105.6)	95.2 ± 24.8	0.724
MMF (L/s)	1.7 ± 1.1	3.4 (1.5 – 4.4)	0.171	1.5 (0.6 – 2.6)	2.6 ± 1.5	0.354
%MMF (predicted, %)	51.2 ± 34.6	95.6 (37.4 – 109.0)	0.284	38.7 (14.7 – 66.9)	74.6 ± 39.0	0.093
Neutrophils (%)	60.4 ± 11.2	61.1 (53.7 – 72.2)	0.833	57.8 (47.3 – 62.2)	64.9 ± 10.2	0.222
Neutrophils (×10^2cells/μL)	39.4 ± 14.3	30.6 (24.8 – 70.4)	> 0.999	30.2 (24.7 – 40.7)	46.9 ± 20.8	0.222
Eosinophils (%)	6.1 (1.6 – 9.8)	1.6 (0.6 – 6.4)	0.354	8.1 (6.1 – 16.5)	1.2 (0.5 – 4.2)	0.011*
Eosinophils (cells/μL)	303.5 (86.8 – 748.0)	80.0 (34.3 – 645.5)	0.354	583.0 (241.1 – 1170.0)	66.0 (31.6 – 274.3)	0.045*
Basophils (%)	0.7 ± 0.5	0.4 (0.2 – 1.1)	0.643	1.1 (0.7 – 1.4)	0.4 ± 0.4	0.019*
Basophils (cells/μL)	45.6 ± 35.8	20.4 (10.0 – 85.9)	0.649	50.6 (36.8 – 97.8)	20.2 (9.4 – 36.6)	0.040*
Lymphocytes (%)	25.7 ± 5.8	29.3 (16.4 – 37.0)	0.803	27.0 (21.2 – 30.4)	26.4 ± 9.1	0.974
Lymphocytes (×10^2cells/μL)	16.6 ± 5.6	15.0 (14.2 – 18.8)	0.943	17.6 (92.6 – 20.8)	17.0 ± 3.5	0.622
Monocytes (%)	5.9 ± 1.9	6.5 (5.3 – 7.9)	0.435	7.0 (5.6 – 8.4)	5.7 ± 1.6	0.284
Monocytes (cells/μL)	377.7 ± 159.4	418.2 (301.3 – 563.6)	0.524	322.0 (265.4 – 625.5)	383.2 ± 131.1	> 0.999
Total IgE (IU/ mL)	217.1 ± 237.8	25.0 (11.5 – 2638.5)	0.435	231.0 (144.0 – 567.0)	23.5 (20.3 – 106.3)	0.045*
Periostin (ng/ mL)	100.6 ± 64.0	45.0 (36.0 – 81.0)	0.182	90.0 (73.5 – 157.5)	43.0 (33.8 – 84.5)	0.051
Tenascin-C (ng/mL)	37.7 ± 17.7	36.8 (20.6 – 156.0)	0.833	29.5 (24.6 – 45.7)	33.0 (23.9 – 70.5)	0.622
Eotaxin-1 (pg/mL)	97.4 ± 56.5	69.4 (64.1 – 143.5)	0.943	90.4 (54.5 – 112.8)	104.8 ± 59.7	0.943
IFN-γ (pg/mL)	12.4 (1.2 – 279.1) (n = 3)	6.5 (5.6 – 7.4) (n = 2)	0.800	6.8 (1.2 – 12.4) (n = 2)	7.4 (5.6 – 279.1) (n = 3)	0.800
IL-5 (pg/mL)	7.4 (0.7 – 9.9) (n = 3)	1.1 (1.0 – 1.7) (n = 3)	0.700	7.4 (0.7 – 9.9) (n = 3)	1.1 (1.0 – 1.7) (n = 3)	0.700
IL-17 (pg/mL)	3.4 (1.3 – 60.1) (n = 3)	1.8 (0.7 – 2.6) (n = 3)	0.400	2.4 (1.3 – 3.4) (n = 2)	2.2 (1.0 – 45.7) (n = 4)	> 0.999

MCP-1 (pg/mL)	612.2 ± 281.4	567.7 (385.6 – 607.5)	0.622	643.8 (300.3 – 936.2)	541.8 ± 180.3	0.622
MIP-1β (pg/mL)	83.0 ± 37.6	54.0 (38.5 – 88.0)	0.435	81.9 (68.1 – 131.8)	61.1 ± 30.1	0.093
RANTES (ng/mL)	30.0 ± 16.7	25.2 (21.5 – 33.7)	0.833	37.8 (20.8 – 49.2)	24.7 ± 11.0	0.222
Th1 cells (% of Th cells, %)	22.3 ± 8.9	16.0 (12.5 – 24.8)	0.435	16.9 (11.8 – 25.3)	22.2 ± 9.0	0.524
Th2 cells (% of Th cells, %)	5.1 ± 2.1	3.7 (1.8 – 6.0)	0.354	5.8 (3.7 – 7.8)	3.9 ± 1.8	0.171
Th17 cells (% of Th cells, %)	5.7 ± 1.2	4.3 (2.4 – 5.4)	0.030*	6.9 (4.8 – 7.2)	4.6 (3.9 – 5.0)	0.019*
Treg cells (% of Th cells, %)	4.8 (3.9 – 6.7)	6.0 (4.6 – 7.4) (n = 4)	0.461	5.4 (4.8 – 8.7) (n = 5)	4.3 (3.8 – 6.7) (n = 7)	0.202
ILC1 (% of ILC cells, %)	66.6 ± 13.5	59.6 (49.6 – 77.5)	0.524	67.2 (52.6 – 72.0)	66.3 ± 17.1	0.943
ILC2 (% of ILC cells, %)	23.6 ± 13.7	24.5 (7.7 – 37.4)	0.943	23.6 (12.4 – 38.9)	22.1 ± 14.4	0.833
ILC3 (% of ILC cells, %)	9.8 ± 4.6	13.1 (6.9 – 22.5)	0.284	9.2 (8.6 – 15.7)	11.6 ± 8.1	0.943
NK cells (% of lymphoid cells, %)	16.5 ± 12.2	6.5 (4.9 – 15.1)	0.222	9.7 (7.8 – 30.6)	7.3 (5.4 – 15.4)	0.354
γδT cells (% of lymphoid cells, %)	3.1 ± 1.3	2.0 (1.4 – 2.9)	0.121	2.6 (2.1 – 3.3)	2.7 ± 1.5	0.591
NKT (% of lymphoid cells, $\times 10^{-3}$%)	1.6 (0.6 – 6.1) (n = 7)	9.1 (4.6 – 17.0) (n = 5)	0.073	3.8 (0.4 – 15.0) (n = 4)	7.5 ± 6.6	0.570
MAIT cells (% of $CD3^+$ cells, %)	1.8 (0.8 – 2.8)	3.5 (0.5 – 4.3)	0.622	1.4 (0.8 – 3.4)	2.3 ± 1.7	> 0.999

For normally distributed data, number quoted is mean ± SD.

For nonparametric variables, number quoted is median (interquartile range).

*$p < 0.05$

Supplementary Figure S1

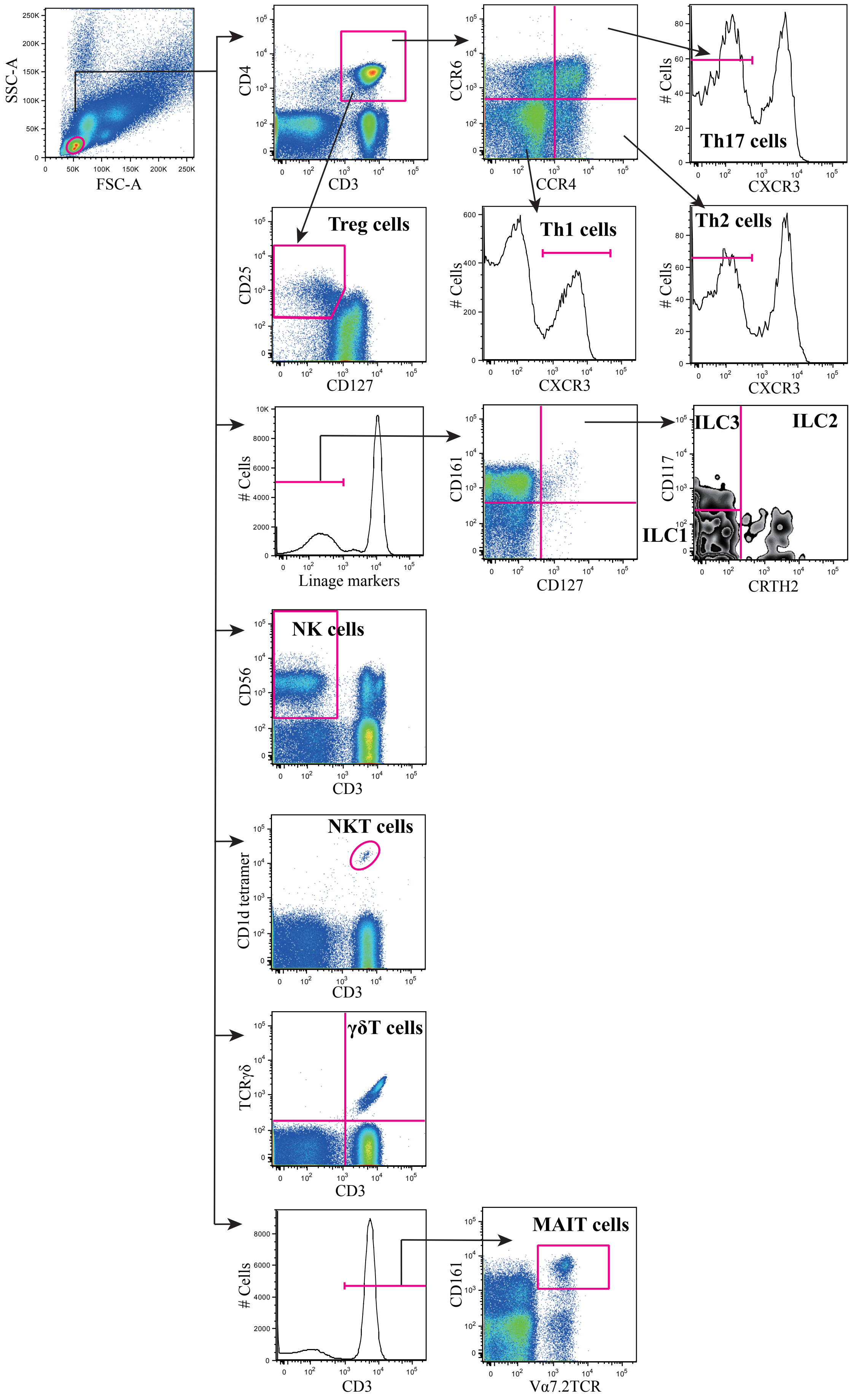

Supplement: Supplementary file 1 [file biomolecules-13-00538-s001.zip › biomolecules-2210158-supplementary.pdf]
